# Boosting Electrochemical Sensing Performances Using Molecularly Imprinted Nanoparticles

**DOI:** 10.3390/bios14070358

**Published:** 2024-07-22

**Authors:** Francesco Gagliani, Tiziano Di Giulio, Muhammad Ibrar Asif, Cosimino Malitesta, Elisabetta Mazzotta

**Affiliations:** Laboratorio di Chimica Analitica, Dipartimento di Scienze e Tecnologie Biologiche e Ambientali (Di.S.Te.B.A.), Università del Salento, Via Monteroni, 73100 Lecce, Italy; francesco.gagliani@unisalento.it (F.G.); tiziano.digiulio@unisalento.it (T.D.G.); muhammadibrar.asif@unisalento.it (M.I.A.); cosimino.malitesta@unisalento.it (C.M.)

**Keywords:** molecularly imprinted nanoparticles, electrochemical sensors, nanoMIPs, electrochemical detection

## Abstract

Nanoparticles of molecularly imprinted polymers (nanoMIPs) combine the excellent recognition ability of imprinted polymers with specific properties related to the nanosize, such as a high surface-to-volume ratio, resulting in highly performing recognition elements with surface-exposed binding sites that promote the interaction with the target and, in turn, binding kinetics. Different synthetic strategies are currently available to produce nanoMIPs, with the possibility to select specific conditions in relation to the nature of monomers/templates and, importantly, to tune the nanoparticle size. The excellent sensing properties, combined with the size, tunability, and flexibility of synthetic protocols applicable to different targets, have enabled the widespread use of nanoMIPs in several applications, including sensors, imaging, and drug delivery. The present review summarizes nanoMIPs applications in sensors, specifically focusing on electrochemical detection, for which nanoMIPs have been mostly applied. After a general survey of the most widely adopted nanoMIP synthetic approaches, the integration of imprinted nanoparticles with electrochemical transducers will be discussed, representing a key step for enabling a reliable and stable sensor response. The mechanisms for electrochemical signal generation will also be compared, followed by an illustration of nanoMIP-based electrochemical sensor employment in several application fields. The high potentialities of nanoMIP-based electrochemical sensors are presented, and possible reasons that still limit their commercialization and issues to be resolved for coupling electrochemical sensing and nanoMIPs in an increasingly widespread daily-use technology are discussed.

## 1. Introduction

The molecular recognition phenomenon, by which molecular structures such as enzymes and antibodies interact with their respective ligands according to specific and selective bonding [1], inspired researchers to build up synthetic receptors in order to avoid the stability issues related to biological molecules outside their physiological environment [2]. The most notable example of biomimetics [3] is well-represented by molecularly imprinted polymers (MIPs), often referred to as antibody mimics or plastic antibodies. MIPs are generated upon the polymerization of functional monomers into a highly cross-linked matrix around the target template, whose removal from the network leaves cavities complementary in shape, size, and chemical functionalities to the target itself, therefore allowing its selective rebinding [4]. The MIP production process involves three main steps, namely, (1) the formation of a pre-polymerization complex between the functional monomers and the template, typically involving non-covalent interactions; (2) the polymerization of functional monomers around the target template, often requiring a cross-linking agent for the stabilization of imprinted sites; and (3) template removal from the polymeric matrix [5]. When compared to their biological counterparts, molecularly imprinted polymers show greater robustness and stability, as well as cost-effective ease of production and long storage lifes [6].

By taking inspiration from nanoscience, researchers focused their attention on synthetic procedures for scaling down the imprinting technology to the nanoscale, combining the excellent MIP recognition properties with benefits related to the nanosize and the high surface-to-volume ratio. Applied to MIPs, the high surface area reflects into binding sites mostly located at the surface of the receptor, resulting in easier access for the target molecule and, in turn, enhanced sensing performances and improved binding kinetics. In addition, the template removal results are more efficient, avoiding template leakage, possibly affecting the rebinding steps with a reduced number of available imprinted cavities and the generation of false results. Furthermore, nanoMIP production can be scaled up with automatic reactors, which is highly suitable for industrial manufacturing [7,8,9,10,11,12]. The possibility of fine-tuning nanoMIPs properties such as size, structure, and solubility, as well as template loading capacity, also through the use of simulation models, refs. [13,14,15], further contributed to the widespread nanoMIPs applications. The flourishing nanoMIPs application in different fields also relies on the possibility of successfully combining most nanoMIPs synthetic approaches with epitope imprinting, enabling the design of imprinted nanoparticles for proteins and other macromolecules, which act as markers for monitoring samples in clinical, environmental, and biotechnological fields. Through this approach, excellent examples of nanoMIPs have been proposed, and their in vivo applications [16] in imaging and drug delivery have been explored [17,18], taking advantage of their nanosize and making them freely eliminable physiologically via the excretory system, along with their high biocompatibility.

Due to their sensing capabilities, nanoMIPs have found wide applications as a recognition element in sensor design, with electrochemical detection representing one of the most explored strategies. Indeed, electrochemical sensors offer several advantages, such as flexibility in design and a wide variety of electrode materials and configurations that allow for customization and optimization for specific applications [19,20,21]. Electrochemical readout is fast, making electrochemical sensors suitable for real-time monitoring applications without extensive sample preparation [20,21]. Another crucial advantage is their portability and miniaturization, which make them ideal for field applications and point-of-care diagnostics. Furthermore, the simplicity of integrating electrochemical sensors with electronic devices facilitates easy data processing, storage, and transmission [19,20,21]. For nanoMIPs coupling with electrochemical sensors, a high number of strategies have been explored to enable robust anchoring with any kind of electrode material for the electrochemical detection of both electroactive and non-electroactive targets, ranging from small molecules to proteins and other macromolecules. The synergistic effect of all these aspects enabled the successful applications of nanoMIP-based electrochemical sensors in food safety analysis [22,23,24], in the detection of environmental pollutants [25,26], and in forensic applications [27].

In the present study, such applications will be reviewed, describing some examples from the last 10 years of literature. After a short overview of the different strategies for nanoMIP synthesis, a description of the most commonly used protocols for nanoMIP integration with electrochemical transducers is reported. Additionally, different strategies for the generation of electrochemical signals in nanoMIP-based sensors are illustrated to provide a comprehensive picture of nanoMIPs effect on boosting electrochemical sensor capabilities.

## 2. NanoMIPs Synthetic Strategies

**Figure 1:**
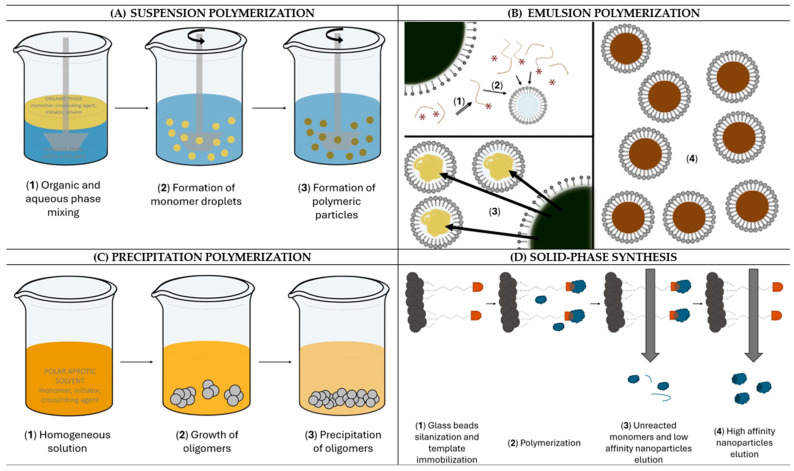
Synthetic strategies of nanoMIPs. (**A**) Suspension polymerization. Adapted from Ref. [29]. (**B**) Emulsion polymerization. (1) Oligomers form in the continuous aqueous phase and (2) migrate inside the micelles, where (3) polymerization continues with monomers diffusing from the droplets until their consumption. Finally, (4) micelles will contain polymeric particles. Adapted from Ref. [30]. (**C**) Precipitation polymerization. Adapted from Ref. [31]. (**D**) Solid-phase synthesis. Adapted from Ref. [32].

**Figure 2:**
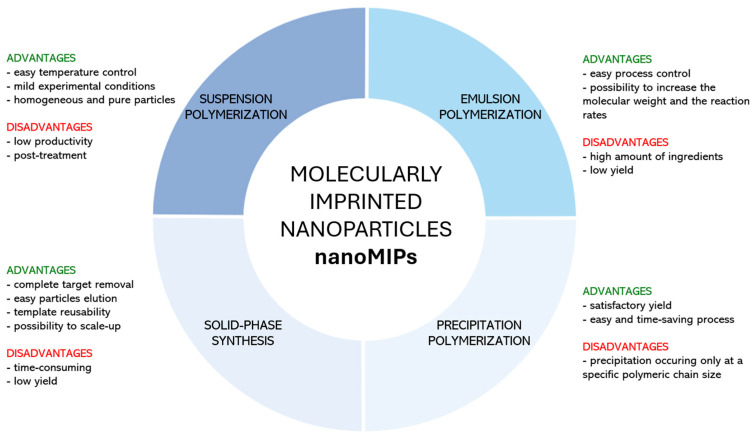
Advantages and disadvantages of nanoMIP synthetic strategies.

The current trend in nanoMIPs production relies on suspension polymerization, emulsion polymerization, precipitation polymerization, and solid-phase synthesis, as depicted in Figure 1. In all cases, particular attention is devoted to the synthesis of water-compatible imprinted nanoparticles, which enables the imprinting of biomolecules that could be damaged by a non-aqueous environment. This is also in good agreement with the green chemistry guidelines [28], which avoid the use of toxic reagents. Figure 2 displays a summary of the advantages and disadvantages of each synthetic approach.

### 2.1. Suspension Polymerization

Suspension polymerization involves an organic phase comprising a monomer, cross-linking agent, solvent, and initiator, and an aqueous continuous phase containing a surface-active agent. When both phases are mixed, the free-radical polymerization starts to remain confined within droplets of the organic phase, leading to the transformation of dispersed liquid droplets into spherical polymeric particles [33]. The presence of water in a continuous phase seems to be particularly important since it can facilitate agitation and promote heat transfer in the whole system [29]. Generally, suspension polymerization seems advantageous because the reaction temperature is easily controllable and the particles show high homogeneity and purity. However, the main drawbacks of this technique are the low productivity and the post-treatment of the resulting material, which is necessary to remove the surface-active agents that can interfere with particle purity [34].

Motaharian et al. prepared nanoparticles imprinted against the pesticide diazinon with a polymerization mixture prepared in chloroform, including template, methacrylic acid (MAA) as a functional monomer, and ethylene glycol dimethacrylate (EGDMA) as a cross-linking agent. Upon the addition of the initiator 2,2-azobisisobutyronitrile (AIBN), the pre-polymerization solution was added to silicon oil, dispersed, and sonicated. Finally, the heating at 65 °C completed polymerization. The imprinted nanoparticles were filtered, and the template molecules were removed by several washings with methanol. The simple procedure led to imprinted nanoparticles with a size less than 100 nm that were mixed with graphite powder to construct a modified carbon paste electrode (CPE), able to rebind the target due to the presence of binding sites nearly at the surface of the nanoparticles [35]. The same polymerization mixture was used by Alizadeh’s group to produce nanosized particles imprinted against the β-adrenergic antagonist timolol. The authors used high-speed mechanical and ultrasonic wave mixing to strongly reduce the droplet size, which was then combined with graphite in order to obtain a modified carbon paste electrode, revealing a strong and reversible target interaction through the imprinted nanoparticles that actually acted as both pre-concentration and sensing elements [36]. Recently, Sullivan et al. developed a microwave-assisted suspension polymerization as an easy and rapid production technique for imprinted nanoparticles involving a toluene organic phase of MAA and steroid compounds as target templates, while the continuous phase consisted of an aqueous solution of polyvinyl alcohol (PVA). Upon the addition of EGDMA and AIBN, the polymerization was carried out at 110 °C in a microwave synthesizer, after which the nanoparticles were collected and subjected to a Soxhlet extraction procedure in methanol/acetic acid to remove the template. By obtaining nanoparticles with a size between 120 and 143 nm and association constants in the micromolar range, the authors claimed the superiority of this strategy over conventional suspension polymerization, since the reagent heating outstandingly minimized the time to reach the activation energy, therefore reducing the nanoparticle synthesis to only 45 min [37].

To produce water-compatible imprinted nanoparticles while ensuring the correct interaction between binding sites and target analytes in an aqueous environment, the *inverse suspension polymerization* was developed by dispersing the aqueous phase, comprising monomer and template molecules, in the organic phase, which contains the surface-active agent able to stabilize the suspension by hydrophilic–lipophilic balance [38]. Prasad and Pathak prepared a multiwalled carbon nanotube-functionalized pencil graphite electrode (MWCNTs-PGE) modified with antitumor dacarbazine-imprinted nanospheres having a size of 340 nm and prepared via inverse suspension polymerization at 70 °C in an aqueous phase of N-acryloylamino butyric acid (ABA), template and cross-linking agent 1,3-diacryloylurea (DAU), and a cyclohexane continuous organic phase containing the stabilizer Span-80, followed by template removal with several washings in methanol. In this study, the use of highly water-soluble ABA as a functional monomer led to electrostatic interaction with the protonated form of the target molecule in an acidic medium, while Span-80 stabilized the imprint molecules at the polymer–aqueous phase interface, resulting in surface-binding sites that were able to fasten the electron transfer kinetics [39].

In suspension polymerization, some difficulties can arise in particle size control due to many factors, such as surface-active agent type, agitation, and the physical properties of both phases. Moreover, the possible interposition of template molecules in the continuous phase could reduce the interactions between monomer and template itself, while the dispersing medium can severely affect the recognition ability of imprinted cavities towards the target [40].

### 2.2. Emulsion Polymerization

Emulsion polymerization involves an aqueous continuous phase and a dispersing organic phase. The organic phase forms droplets that are stabilized by a water-soluble surfactant and contain a large amount of water-insoluble monomer. However, a few monomer molecules are present in the continuous phase, where they react with a water-soluble initiator. As a consequence, oligomers are formed and stabilized by micelles. The polymerization continues inside the micelles upon the diffusion of monomers from the droplets until their consumption [30]. Generally, the surfactant can be anionic or cationic, depending on the initiator, so particle colloidal electrostatic stabilization is established [41]. The main advantages of emulsion polymerization are the easy process control, the possibility to increase the molecular weight of the resulting polymer, and the reaction rates. However, it requires a large amount of ingredients, which are quite difficult to remove, thus leading to impurities, and the yield is definitely reduced by the presence of water [42].

Specifically, nanoMIPs are usually prepared by *miniemulsion*, where liquid/liquid emulsions consisting of 50–500 nm droplets are present [43]. Miniemulsion polymerization requires the presence of a costabilizer to retard the diffusion from monomer droplets [44], while the size of monomer droplets acting as “nanoreactors” is kept constant by high-pressure homogenizers, ultrasonication, and other shear devices [45,46,47,48]. Farzaneh et al. used miniemulsion polymerization through a non-covalent mechanism to produce nanoMIPs with a size less than 100 nm for solid-phase extraction combined with HPLC and controlled release in the central nervous system of the antipsychotic drug olanzapine. The polymerization was carried out at 70 °C, involving a hexadecane organic phase of template, MAA, EGDMA, and AIBN and an aqueous continuous phase of sodium dodecyl sulfate (SDS) as surfactant. After the reaction, the surfactant was removed by dialysis, while the template extraction was performed by washing with methanol/acetic acid. The authors optimized the ratio between functional monomer and template and the pH of the medium, providing the greatest drug loading, therefore assessing the production of highly efficient imprinted nanoparticles suitable for the sensitive extraction and controlled release of olanzapine with low cytotoxicity on a fibroblast cell line [44]. Poufarzib et al. prepared water-compatible, imprinted nanosized particles for the extraction and purification of the non-nucleoside reverse transcriptase inhibitor efavirenz in human serum and urine. A mixture of MAA, the cross-linking agent trimethylolpropane trimethacrylate (TRIM), and the hydrophobic agent hexadecane was formed, followed by the addition of template and AIBN. Subsequently, the final mixture was mixed with a SDS dispersing phase, and the polymerization was carried out at 70 °C. Collection and dialyses of the as-produced nanoparticles were performed, followed by methanol/acetic acid washings to remove the target template. The procedure resulted in the production of spherical nanoparticles with a size between 217 and 253 nm. The high surface-to-volume ratio enabled pronounced accessibility to the binding sites, thus fastening the analyte equilibration in HPLC target detection by using imprinted nanoparticles as selective sorbents [49]. The miniemulsion method was also used by Esfandyari-Manesh’s group to produce spherical molecularly imprinted nanoparticles with a size of 181 nm as delivery carriers for the anti-neoplastic drug paclitaxel in tumor cell lines. The synthesis consisted of a chloroform organic phase comprising the target template, MAA, and methyl methacrylate (MMA) as functional monomers and EGDMA and hexadecane as hydrophobic agents, which were injected in a syringe containing a SDS dispersing phase. The polymerization was carried out at 70 °C, after which the template was removed by washing several times with methanol/acetic acid. The imprinted nanoparticles were activated by EDC and then conjugated with polyethylene glycol (PEG)–folic acid (FA) to obtain the ternary system MIP-PEG-FA being internalized by the cell lines involved in this study [50]. Recently, Ozgur et al. resorted to a two-phase miniemulsion polymerization to produce nanoparticles against the dye amaranth. The procedure involved a first aqueous phase of SDS, sodium bicarbonate, and PVA, which was mixed with the oil phase of the pre-polymerization complex, comprising template, methacrylic acid, acrylamide, 2-hydroxyethyl methacrylate, and ethylene glycol dimethacrylate. Then, a second aqueous phase of SDS and PVA was added, followed by sodium bisulfite and ammonium persulfate as initiators. The polymerization was carried out at 40 °C under agitation, resulting in the formation of spherical nanoparticles with a size of about 70 nm, which were then used for the construction of a sensing system based on surface plasmon resonance that maintained its stability for 6 months [51].

As for suspension polymerization, emulsion polymerization can also be carried out by inverting the two phases in the so-called *inverse emulsion polymerization*, enabling the polymerization of water-soluble functional monomers in a shorter time [43]. Indeed, Weber et al. imprinted nanoparticles against penicillin G (PenG) to be used as a sensing layer for the target optical detection in buffer media. The aqueous dispersing phase consisted of N-(2-aminoethyl) methacrylamide hydrochloride (NAEMA) as a functional monomer, ethylenebisacrylamide (EBA) as a cross-linking agent, and the template, while the cyclohexane continuous phase comprised Span-80 as a surfactant and AIBN as an initiator. The polymerization was carried out in the aqueous droplets at 55 °C upon the combination of the two phases. The as-produced nanoparticles showed an average size of 206 nm, which doubled when the nanoparticles were functionalized with an azide group involved in the click chemistry reaction for their covalent immobilization onto the transducer surface. The authors stated that the as-produced nanoparticles possessed an outstanding target rebinding capacity in the concentration range of 0.0015–0.0195 mol/L without reaching any saturation state, therefore assuring the possible detection of penicillin G at higher concentrations [52].

As an alternative to conventional emulsion polymerization, Dvorakova et al. proposed the so-called *nonaqueous emulsion*, consisting of a simple and high-yielding strategy based on the replacement of water with *n*-hexane as a continuous phase, while the dispersing phase containing the template, functional monomer, and radical initiator was based on dimethylformamide (DMF). This strategy led to the production of 100 nm imprinted particles with a greater imprinting effect compared to water-based emulsion polymerization [53,54].

It should be highlighted that a continuous phase based on water could lead to a reduced interaction between monomer and template, thus affecting the imprinting process. Moreover, surfactants show some difficulties in their complete removal, which could constitute a problem for in vivo application due to the denaturing power of such compounds towards proteins [17].

### 2.3. Precipitation Polymerization

Precipitation polymerization is a very simple yet effective method to produce polymeric particles involving the initial solubilization of monomer, cross-linking agent, and initiator molecules in a polar aprotic solvent, where oligomers precipitate due to their lower solubility [55,56]. The procedure does not require a surfactant, avoiding any impurity issue, while meticulous control of polymerization conditions allows to finely tune the size and morphology of particles [56]. As proposed by Ye et al., monomer molecules can be pre-polymerized around the target template and then transferred into a poor solvent, leading to the precipitation of particles, from which the template is removed. This alternative strategy was adopted by Schirhagl et al., who dissolved template, cross-linker, and functional monomer in water, where the polymerization occurred under UV light. Afterwards, an aliquot of the solution was injected into acetonitrile, leading to the precipitation of spherical nanoparticles with sizes in the range of 15–700 nm [57,58]. Precipitation polymerization, which can be initiated either by UV radiation or temperature, permits the production of satisfactory particle yields with an easy and time-saving procedure. However, the precipitation of particles occurs only when the polymeric chains are large enough so that they become insoluble in the reaction mixture [59].

Contin’s group produced nanoMIPs against coenzyme Q10 (CoQ10) at 70 °C in a water/acetonitrile mixture, involving CoQ10 as a dummy template, MAA, EGDMA, and potassium persulfate (KPS). The procedure led to nanoparticles being used as sorbents in a CoQ10 dispersive extraction, leveraging the affinity of nanoparticles for the target and enabling the use of only 1 mg of the sorbent for the process [60]. Khosrokhavar et al. imprinted nanoparticles against the antidepressant drug sertraline, involving a chloroform solution of template, MAA, EGDMA, and AIBN. The polymerization was carried out at 65 °C, after which the template was removed by washing in methanol/acetic acid, resulting in imprinted nanoparticles of about 50 nm that were then combined with graphene in order to modify screen-printed carbon electrodes for the electrochemical detection of sertraline, highlighting the ability of imprinted nanoparticles to bind the target [61]. Spherical and porous nanoparticles having a size in the range of 60–300 nm were produced by Mohebali’s group for the antidepressant amitriptyline hydrochloride (AT). The results showed that nanoparticles produced at 60 °C in a chloroform/amyl acetate solution of template molecules MAA, TRIM, and AIBN displayed higher absorption capacities, resulting in a controlled and sustained release of the drug due to binding site enhancement by the polar aprotic solvent amyl acetate [62].

Precipitation polymerization enables a facile synthesis for molecularly imprinted nanoparticles, although some drawbacks, such as low productivity, monomer-template reduced interactions due to high dilutions, and polymerization parameter optimization for each type of system, may limit its use. However, an improvement in the interaction between the monomer and the template can result from the addition of a higher amount of template [17,63,64].

### 2.4. Solid-Phase Synthesis

An alternative widely used approach for nanoMIP production is inspired by the principle of solid-phase synthesis and exploits the preliminary target template immobilization onto a solid support. The polymerization is then initiated, enabling the formation of molecularly imprinted nanoparticles that are eluted to collect high-affinity nanoMIPs after a first elution aimed at removing non-reacted monomers and low-affinity polymer nanoparticles. The solid support generally consists of glass beads exposing silanol groups suitable for subsequent template anchoring by exploiting a specific coupling chemistry in relation to the nature of the template and available functional groups [65]. By this approach, nanoMIPs free from templates are directly obtained after the elution step, thus avoiding the tedious and long washing step. Moreover, the target template immobilized on the solid support can be reused for several synthesis processes, flattening the costs associated with nanoMIP production. Lastly, as demonstrated by Poma et al., it is possible to scale up production with an automatic reactor, improving industrial manufacturing [66]. However, compared to other methods, it can be time-consuming, given that the solid phase must be prepared in the proper way. Additionally, low yields of the resulting imprinted nanoparticles are sometimes reported [37].

The solid-phase synthesis of nanoMIPs was developed almost in parallel in the second decade of the 2000s by two distinct research groups that adopted specific conditions.

Piletsky’s group proposed an approach involving template covalent immobilization onto the solid phase, followed by the synthesis of nanoMIPs through the polymerization of water-soluble functional monomers to produce nanoparticles against peptides and proteins, therefore preserving their structures in an aqueous environment, or MAA and TRIM in organic solvents, more suitable for the imprinting of small molecules. The polymerization mixture must be purged with nitrogen to eliminate oxygen, whose presence can inhibit the process. Then, the synthesis can be initiated with persulfate for polymerization in water or by UV radiation, as in the case of organic solvents. After the production, unreacted monomers and low-affinity nanoparticles are removed with room-temperature water, while high-affinity nanoMIPs are eluted at higher temperatures and then subjected to a dialysis step in order to purify and concentrate the sample [32].

In the approach developed by Haupt’s group, the synthesis typically involves KPS as a free radical polymerization initiator and the polymerization of a thermoresponsive functional monomer, N-isopropylacrylamide (NIPAm), providing nanoparticles with thermoresponsiveness. Indeed, NIPAm is subjected to a volume-phase transition at its lower critical solution temperature (LCST), meaning that it goes from a swollen hydrated state at 37 °C to a shrunken dehydrated state at 4 °C [67,68]. The resulting nanoparticles thus acquire a collapsed state around the template at 37 °C, while the elution at a lower temperature (4 °C) allows their swelling and thus their detachment from the immobilized template. This feature showed to be particularly suitable for nanoMIPs application in physiological conditions, where they will favorably bind the target [69].

Garcia-Mutio et al. used the solid-phase approach to produce nanoparticles in the range of 128–163 nm against the phenolic compound 4-ethylphenol. The authors functionalized glass beads as solid support first with N-[3-(trimethoxysilyl)propyl]ethylenediamine (EDPTMS) and then with glutaraldehyde, providing a ketonic group able to react with the primary amine of template analogue tyramine, allowing its immobilization onto glass beads. Any unreacted aldehyde group was blocked with ethanolamine, and the polymerization was carried out in organic solvent via UV irradiation in the presence of different functional monomers in order to obtain the most performing nanoparticles. The ones produced by using 4-vinyl pyridine (4VP) as a functional monomer proved to be the most selective and were therefore used for sensor fabrication [70]. Crapnell et al. imprinted nanoparticles having a size below 300 nm by using heart-fatty acid binding protein (H-FABP) and interleukin receptor ST2 as protein templates. The dissociation constants were absolutely comparable to those of commercially available antibodies, however, promising results were obtained in the simultaneous detection of both analytes, either in buffer or spiked serum, with negligible cross-selectivity, proving the potential application of such a nanosized system in point-of-care applications [71]. Kassem et al. reported the synthesis of imprinted nanoparticles towards trypsin in the presence of fluorescein acrylamide, allowing them to observe biodistribution and cytotoxicity in rat liver, spleen, intestine, and brain, therefore showing the suitability of such imprinted nanosized materials as bioimaging tools [16]. Cavalera et al. analyzed the effects of different experimental conditions in the solid-phase synthesis of imprinted nanoparticles against the antibiotic ciprofloxacin. In particular, the authors studied the functionalization of glass beads followed by template immobilization, the monomeric composition of the polymerization mixture, namely, N,N′-methylenebisacrylamide and ethylene dimethacrylate/trimethylolpropane trimethacrylate, and the solvent nature, water, or acetonitrile. The results showed that the nanoparticle populations were differentiated in terms of size, ranging from 147 to 255 nm, mainly due to the presence of a glutaraldehyde chain acting as a spacer arm between glass beads and growing polymeric structures. NanoMIPs prepared in water showed strong binding in an acidic environment due to hydrogen interactions promoted by protonated carboxylic groups of the polymer, while acetonitrile-produced nanoMIPs resulted in the strongest binding at neutral or basic pH values, thanks to the interaction between the deprotonated and negatively charged methacrylic acid and the positively charged secondary nitrogen of ciprofloxacin [72]. Some authors also explored the possibility of replacing glass beads as solid support with two-dimensional surface quartz chips onto which the target template indole-3-butyric acid, preliminary silylated and therefore indicated as IBA-APTES, was immobilized through stable covalent bonds. The polymerization was then carried out under UV irradiation at 4 °C in an acetonitrile solution, leading to uniform and selectively imprinted nanoparticles with a size of 80–100 nm. The use of quartz chips allowed the authors to measure the contact angle after IBA-APTES immobilization, claiming successful template grafting that was further confirmed by XPS analysis, IR spectrometry, UV spectrometry, and fluorescence spectra [65].

Haupt’s group pioneering study involved the immobilization of trypsin inhibitor *p*-aminobenzamidine (PAB) onto the solid support, its complex formation with the target template trypsin, and the synthesis of highly specific and selective nanoMIPs [73]. Lately, the same inhibitor was successfully used to imprint nanoparticles against kallikrein, remarking the versatility of the strategy. Moreover, nanoMIPs against trypsin, kallikrein, and RNase A were produced by using Cu^2+^-IDA as a metal chelate bound to the solid support, leveraging the affinity of copper ions for surface-exposed histidine moieties possessed by most proteins. Although metal chelates represented a promising strategy, nanoMIPs produced in the presence of PAB showed improved binding affinities, selectivity, and stability over a 6-month storage period at 4 °C [68]. In contrast, high-affinity nanoparticles for adenosine 5′-monophosphate (AMP) were produced by exploiting the interaction of iron ions with phosphate groups; therefore, Fe^3+^-IDA metal chelate was used as an affinity ligand for nanoMIPs production, while PAB as an affinity ligand involved π–π stacking with the AMP nucleobase, leading to unspecific nanoparticles. This study stated that a Fe^3+^-chelate can potentially be used for any target molecule bearing phosphate groups [74].

A winning strategy consists in combining nanoMIPs solid-phase synthesis with the so-called *epitope imprinting approach*, which consists in the use of protein epitopes as templates instead of the whole protein, which is revealed to be highly suitable for the imprinting of several proteins. The use of an epitope as a template ensures several advantages compared to whole proteins. First, their elementary structure is not prone to three-dimensional conformations; therefore, the formation of homogenous binding sites is guaranteed, improving nanoMIP sensitivity and selectivity [75]. In addition, epitopes can be easily synthesized, reducing the cost associated with their use as templates [76], and finally, they are not susceptible to organic solvents.

Moczko et al. made a comparison between nanoparticles imprinted either against the Fc domain of human IgG or the end peptide epitope, claiming that, although both nanoparticle populations were highly selective toward antibodies, the use of epitope as a template was more economically affordable while providing at the same time high affinity for the resulting synthetic receptors [77]. Herrera León et al. produced nanoMIPs against a protein loop fragment of tumor necrosis factor α (TNF-α) exposed to the solvent, able to bind TNF-α with outstanding affinity and prevent binding to its receptor TNFR1, downregulating the secretion of pro-inflammatory cytokines in a macrophage model [78]. The epitope imprinting approach has also been coupled with the solid-phase synthesis of nanoparticles in the presence of fluorescent monomers, thus obtaining fluorescent nanoMIPs for imaging assays. Medina Rangel et al. produced nanoparticles against a hyaluronic acid substructure, namely, D-glucuronic acid (GlcA), by incorporating a rhodamine fluorescent monomer in the polymeric structure. The resulting nanoparticles were able to detect hyaluronic acid both in extracellular and intracellular spaces with a nanomolar dissociation constant [79].

## 3. Integration of NanoMIPs with Electrochemical Transducers for Sensing Purposes

For the development of electrochemical sensors using nanoMIPs, two distinct steps of nanoMIP synthesis and further immobilization onto the electrode surface are required, thus enabling the separate optimization of the two phases. A complete nanoparticle characterization in terms of size, chemical structure, and morphology can be thus performed, providing useful information to properly select the strategy for their anchoring to the electrode. Along with nanoMIP immobilization onto the electrode surface, which is the most commonly adopted approach, their entrapment within electrode material is also proposed as a way for producing nanoMIP-modified electrodes for electrochemical sensor design. Examples of the two strategies will be presented in this section.

### 3.1. NanoMIPs on the Electrode Surface

#### 3.1.1. NanoMIPs Anchored to the Electrode Surface with Polymeric Films

For integrating nanoMIPs to the electrode, a simple approach consists in physically entrapping nanoparticles in polymeric films, which can be preliminary formed (as Nafion, agarose, or poly(vinyl chloride) (PVC)) or polymerized in the presence of nanoparticles (as electropolymerized films). In the first case, drop-casting, spin coating, or sol–gel techniques can be used. Depending on the specific approach, nanoMIPs can be dispersed in a polymer solution and co-deposited on the electrode, or the polymeric layer can be used for coating the electrode, then promoting the subsequent anchoring of the nanoparticles, or, vice versa, nanoparticles can be deposited first on the bare electrode, then using the polymeric film as a “protective” layer to prevent nanoparticle detachment from the electrode surface.

The entrapment of nanoMIPs in agarose matrix has been explored in a seminal study from Mosbach’s group [64], after which it has been successfully applied to nanoMIPs [80,81]. By this method, nanoMIPs are added to an agarose solution to obtain a slurry, which is then dropped on the electrode surface and dried at room temperature or by thermal [82] or infrared-light treatment until a solid membrane is formed [83]. While entrapment using agarose ensures nanoMIP adhesion on the electrode surface, an issue is related to the agarose effect in limiting analyte diffusions towards nanoMIPs. This leads to slow binding site accessibility and low binding capacity [84]. Agarose can also affect target elution after rebinding, thus affecting sensor re-usability [85]. Nafion is another polymeric material used for this purpose [86]. Typically, nanoMIPs are suspended in a Nafion solution in water or ethanol, deposited on the electrode surface by drop-casting or spin-coating, and then dried. Using this approach, Neto et al. [87] developed a MIP-based amperometric sensor able to detect 4-aminophenol. The authors employed Nafion as a binder to improve the anchoring of nanoMIPs on the electrode surface without compromising sensor sensitivity, benefiting from Nafion’s high ionic conductivity. According to Xia et al., Nafion not only improves the anchoring of nanoMIPs but also enhances the rebinding process through electrostatic interactions with the sulfonic acid groups and fluorine atoms [88,89]. Mazzotta et al. compared two different methods for coupling vancomycin-imprinted nanoparticles with glassy carbon electrodes (GCEs): drop-deposition onto the electrode surface, allowing the solvent to evaporate, and nanoMIP self-assembly on a Nafion-coated GCE. To this end, Nafion was first deposited on the GCE electrode, which was then immersed in an acidic solution of nanoMIPs, exploiting the electrostatic interaction between negatively charged sulfonic groups of Nafion and protonated amino groups of nanoparticles, which were ad hoc introduced in their composition. It was observed that the use of Nafion for nanoparticle self-assembly improved the stability and performance of the sensor in vancomycin detection [90].

PVC can also be used as a matrix to incorporate nanoMIPs. In a typical example, Alizadeh et al. developed a potentiometric sensor for lactic acid using a 2 mm diameter graphite rod as the working electrode, functionalized with nanoMIPs synthesized via precipitation polymerization. The graphite rod was dipped into a mixture containing PVC and nanoMIPs for a few seconds to coat it. The membrane-coated electrode was then dried for 24 h at room temperature [91]. The sol–gel approach has been used by several authors to obtain nanoMIP-based electrochemical sensors for different targets [92]. It involves converting a colloidal suspension of an alkoxide precursor into a gel containing nanoMIPs. This process starts with a “sol”, which is gradually transformed into a “gel” through hydrolysis, polycondensation, and drying, effectively integrating nanoMIPs onto the electrode surface by trapping them in an inorganic framework without affecting the permeability of the target toward the nanoMIPs [93]. Del Valle’s group presented a sol–gel-based method for immobilization of MIP nanoparticles (~820 nm) for theophylline. A sol–gel solution was first prepared by mixing tetraethyl orthosilane (TEOS) and graphite microparticles in acidic medium (after HCl addition), vigorously stirred, and then rested to initiate the syneresis stage. Later, nanoMIPs prepared by precipitation-polymerization were added to this solution to obtain a composite material that was deposited on the surface of an epoxy-graphite electrode. At the end, the electrode was dried at 5 °C for an overnight step, and a sensitive membrane with a thickness of 200–300 μm was obtained [85]. Bakas et al. proposed the entrapment of nanoMIPs on screen-printed carbon electrodes via a sol–gel method to obtain an impedimetric sensor for the organophosphorous insecticide methidathion. First, the sol–gel solution was prepared by mixing tetramethoxysilane (TMOS) and PEG in an acidic medium. Then, nanoMIPs were added to the sol–gel solution, and then the obtained mixture was deposited on the surface of the working electrode and allowed to dry at room temperature to obtain the sensitive layer [93].

The use of preformed polymers or gels entrapping nanoMIPs can indeed lead to some drawbacks, such as non-specific interactions with the support layer, especially when working with complex matrices [94], or a reduced contact between nanoMIPs and the transducer surfaces [95], which is essential for preserving sensor features and maintaining sensitivity. Furthermore, balancing the amount of nanoMIPs within the support material is strongly required and often requires extensive experimental study to determine the optimal conditions.

As an alternative to the use of preformed polymers for nanoMIPs anchoring to the electrode, the growth of a polymeric layer in the presence of nanoparticles can be performed, with the obvious advantage of controlling/selecting specific polymer properties (thickness, density, and morphology) based on nanoMIPs to integrate within. In this sense, electropolymerization is revealed to be a highly suitable approach, as by adjusting specific experimental parameters, such as the amount of circulating charge, it is possible to modulate film features to align with those of nanoMIPs. Indeed, using this approach, thin layers can be obtained, which can contribute to accelerating the rebinding process [22]. One pioneering example of this strategy, although referred to as nanoMIPs, was proposed by Malitesta’s group, which developed an electrochemical sensor by immobilizing ephedrine-imprinted nanoMIPs within an electrosynthesized and overoxidized polypyrrole (PPy) matrix onto the surface of a glassy carbon electrode [96]. A similar approach was proposed almost in parallel by Ho et al., who used poly(3,4-ethylenedioxythiophene) (PEDOT) to anchor morphine-imprinted nanoMIPs onto indium-tin oxide (ITO) electrodes, ultimately used as amperometric sensors [97]. Granado et al. developed a voltammetric sensor for diphenylamine using a gold electrode with nanoMIPs entrapped within an electrosynthesized PEDOT membrane. Interestingly, three different methodologies were used to deposit the PEDOT/nanoMIPs on the gold electrode: (i) by performing cyclic potential scans, (ii) by fixing the potential at 1.2 V (potentiostatic), and (iii) by fixing the current through the electrode (galvanostatic). Better results in terms of target permeability were obtained with PEDOT deposited under galvanostatic conditions. Moreover, to optimize polymer thickness, different electropolymerization times were considered: 1, 2, 4, and 8 min. More satisfying results in terms of sensor sensitivity and limit of detection were obtained after 1 min of electropolymerization. The sensor was effectively applied to quantify diphenylamine in spiked apple juice samples [98].

**Figure 3:**
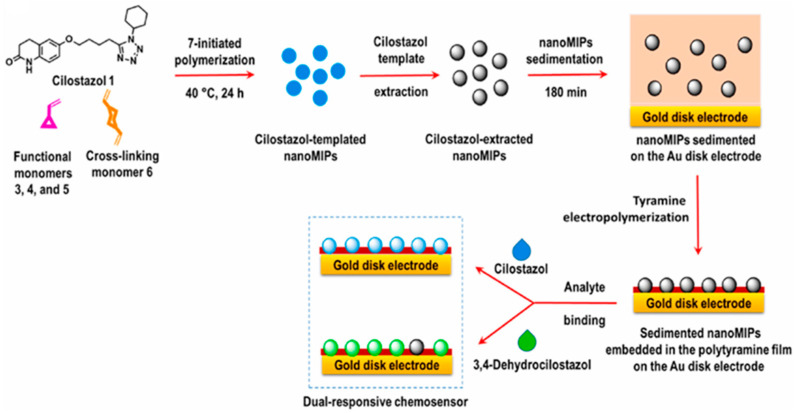
Preparation of an electrochemical sensor for cilostazol detection, where nanoMIPs are embedded in a polytyramine film. Adapted from Ref. [99].

Also, electropolymerized polytyramine was successfully used to this aim in an interesting study by Gonzato et al. [99], who proposed to immobilize nanoMIPs by electrodepositing a mixture containing tyramine and nanoMIPs on gold electrodes via cyclic voltammetry (CV) to obtain an electrochemical sensor for cilostazol detection in human plasma (Figure 3). As stated by the same authors, optimizing the electrode surface coverage with nanoMIPs embedded in the polytyramine film is crucial for sensor performance. Complete coverage of nanoMIPs can indeed hinder the analyte diffusion to the nanoMIP imprinted cavities. In contrast, low film thickness can affect the mechanical stability of the nanoMIPs on the electrode surface. The authors optimized the polymer deposition to achieve a film thickness comparable to that of the nanoMIPs, as evidenced by AFM measurements.

An unconventional but interesting method was proposed by Singh’s group, which developed an electrochemical quartz crystal microbalance sensor to detect *Mycobacterium leprae* bacteria through its epitope sequence. Multiple monomers, including 3-sulphopropyl methacrylate potassium salt, benzyl methacrylate, and 4-aminothiophenol, were used for the synthesis of nanoMIPs in the presence of the bacterial epitope as a template, which were then deposited on a gold-coated quartz electrode via the electropolymerizable 4-aminothiophenol moieties grafted onto the nanoMIPs surface. This approach allows for the direct anchoring of nanoMIPs onto the transducer surface using nanoMIP solutions without the need for additional monomers or reagents. Moreover, there is no risk of creating excessively thick or thin layers, as the optimal mass loading of nanoMIPs on the electrode is indirectly indicated by the stability of the recorded currents. Impedimetric measurements and contact angle analyses indirectly demonstrated the successful anchoring of nanoMIPs [100].

#### 3.1.2. NanoMIPs Anchoring to the Electrode Surface via Coupling Chemistry

Another method for coupling nanoMIPs with the surface of electrochemical transducers involves their anchoring through coupling chemistry procedures [22,86]. Various options are available, depending on the electrode material.

**Figure 4:**
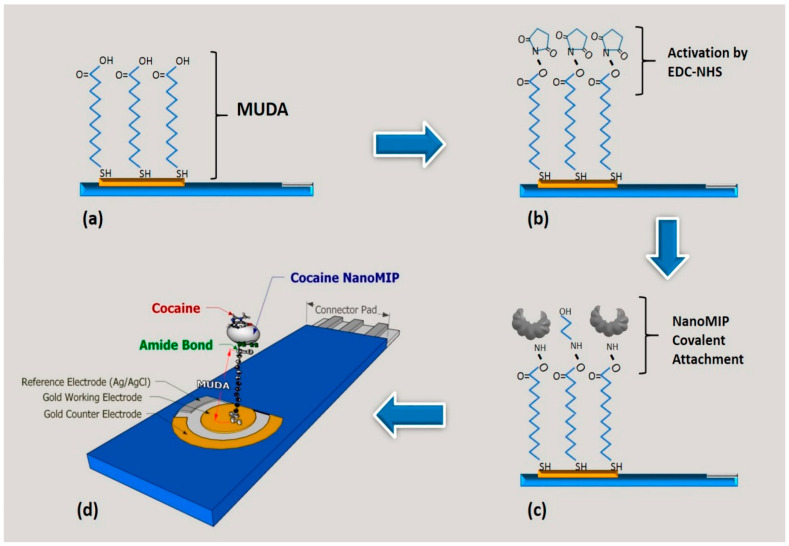
Development of an electrochemical sensor for cocaine detection, where nanoMIPs are anchored to the gold electrode surface through amine coupling chemistry. (**a**) Formation of a self-assembly monolayer of MUDA on gold surface. (**b**) Activation of MUDA carboxyl groups by EDC/NHS. (**c**) NanoMIPs attachment on gold surface by EDC/NHS-mediated reaction between nanoMIPs amino groups and MUDA carboxyl groups. (**d**) Final configuration of the as-functionalized sensor. Adapted from Ref. [101].

In the case of gold electrodes, self-assembly monolayer (SAM) formation is often performed to expose desired functionalities, enabling the subsequent covalent linkage with nanoMIPs, either directly or after intermediate activation steps. SAM of 11-mercaptodecanoic acid (MUDA) is typically used for modifying gold electrodes, followed by exposure to a mixture of EDC/NHS (1-ethyl−3-[dimethylaminopropyl]carbodiimide/N-hydroxysuccinimide) to activate the carboxylic groups of MUDA, thus enabling the attachment of nanoMIPs via amine coupling chemistry. In a classic study, D’Aurelio et al. developed an electrochemical sensor for cocaine with the ability to detect cocaine in a linear range between 100 pg mL^−1^ and 50 ng mL^−1^ and a limit of detection of 0.24 ng mL^−1^ via impedimetric measurements (Figure 4) [101]. All functionalization steps were characterized using electrochemical impedance spectroscopy (EIS), and the attachment of nanoMIPs to the electrode surface was monitored by atomic force microscopy (AFM). During their synthesis, nanoMIPs were functionalized with primary amino groups, enabling their covalent attachment to the sensor surface, although direct chemical characterization of the nanoparticles was not provided. After anchoring the nanoMIPs, unreacted activated carboxylic groups were passivated with ethanolamine to prevent non-specific adsorption. Interestingly, the authors also investigated several blocking agents in combination with ethanolamine, such as (i) BSA and Tween 20, (ii) milk proteins, and (iii) PVA, in various ratios. For each combination, the impedimetric signal was monitored, and the use of ethanolamine alone was selected, resulting in the least resistive system. EIS measurements confirmed nanoMIPs anchoring to the electrode surface, although their adsorption cannot be excluded, as claimed by the authors.

The EDC-NHS coupling chemistry was also exploited in combination with other SAMs on gold electrodes, such as cysteamine, used, for instance, for anchoring nanoMIPs for glucose, trypsin, and paracetamol detection [102], and lipoic acid, used for anchoring nanoMIPs for 4-ethylphenol (4EP) [70] and trypsin [103]. When this coupling chemistry is performed, the successful electrode functionalization steps are typically characterized by EIS and CV monitoring changes in the permeability of a redox probe towards the sensor surface [101]. The anchoring of nanoMIPs on the electrode surface can affect the signal differently based on their electrochemical properties, with resistive nanoMIPs increasing impedance and nanoMIPs involved in electrochemical-assisted electron exchange determining an increase in currents recorded in CV [104].

In an interesting study, Garcia-Mutio et al. examined the influence of the SAM agent used for anchoring nanoMIPs on gold electrode surfaces, monitoring the process with EIS and CV. Specifically, they compared the performance of MUDA and lipoic acid, observing that both are effective for anchoring nanoMIPs. However, when MUDA is used, higher impedance values are recorded compared to those obtained using lipoic acid. This is because MUDA is a long-chain alkanethiol that forms highly ordered monolayers, creating a barrier for electron transfer. Additionally, they studied the impact of using ethanolamine as a blocking agent after nanoMIP anchoring. They found that ethanolamine leads to very high charge transfer resistance, determining much higher insulation of the gold surface compared to the system without a capping agent. Since the sensor sensitivity was significantly reduced in the presence of the capping agent, the authors opted to use the sensors without ethanolamine [70].

In another study, Di Masi et al. investigated the influence of different methods for grafting SAM layers on the nanoMIP anchoring process, monitoring the results with EIS. They formed self-assembled monolayers of lipoic acid by both self-deposition (immersing the gold electrode in a SAM agent solution overnight) and electrochemical deposition via the chronoamperometric technique. In both cases, an increase in electron transfer resistance was observed, obviously attributed to the presence of the SAM layers hindering electron transfer. The assembly of nanoMIPs onto both types of SAM layers resulted in an additional increase in impedance, suggesting a further reduction of interfacial electron transfer. However, nanoMIP immobilization was more pronounced when using the electrodeposited SAM layer, possibly due to more effective and reproducible coverage of the gold electrode surfaces under controlled applied potential compared to conventional passive adsorption [103].

In an interesting study, Garcia-Cruz et al. developed an electrochemical sensor for detecting insulin in human plasma using screen-printed platinum electrodes (SPPEs). In this case, electroactive molecularly imprinted polymer nanoparticles were obtained through solid-phase synthesis by incorporating an electrochemically active ferrocene monomer (ferrocenylmethyl methacrylate, FcMMA) into the polymerization mixture. After synthesis, the nanoMIPs were anchored on screen-printed platinum electrodes using coupling chemistry involving 3-aminopropyltriethoxysilane (APTES) and glutaraldehyde as linkers. The SPPEs were activated with nitrogen plasma and then silanized by incubation in an APTES solution. Subsequently, a mixture of nanoMIP and glutaraldehyde was drop-cast onto the working surface of the electrode. Again, the amino groups of the nanoMIPs were used for their anchoring by the amine coupling reaction. In this case, the presence of amino groups in nanoMIP composition was confirmed by Fourier-transform infrared (FT-IR) analysis. The nanoMIPs anchoring onto the electrodes by coupling chemistry procedures resulted in sensors with good kinetics and target affinity. Additionally, nanoparticle stability on the electrode was satisfying, allowing the sensor to be stored and used for several months without special care [105].

From the examples listed above, it is evident that coupling chemistry for anchoring nanoMIPs on the electrode surface typically relies on the use of aminic moieties in nanoparticle structure, enabling covalent bonding with carboxylic or aldehydic groups. To this end, the chemical characterization of nanoMIPs to confirm the presence of such functional groups available for coupling protocols could be highly beneficial, although it is only rarely reported. Moreover, the use of a blocking agent in this application is controversial. While it is necessary to passivate residual active functionalities after nanoMIP anchoring, its use can make electrochemical systems extremely resistive and difficult to use, which prevents a uniform position in the literature about its use in such applications.

In an intriguing example, to modify the gold electrode surface for nanoMIPs, anchoring the electropolymerization of tyramine was initially performed by CV, introducing free primary amino groups on the electrode surface. Subsequently, the electrode was immersed in a glutaraldehyde solution to induce a coupling reaction, leaving one free aldehyde group. The electrodes were then incubated in a nanoMIPs aqueous solution for anchoring, followed by immersion in a 1-dodecanethiol solution to passivate the bare gold surface. This approach was utilized to obtain capacitive sensors for tetrahydrocannabinol (THC) and a protein (trypsin) [106].

A promising yet underexplored approach involves anchoring nanoMIPs onto the surface of electrodes through electrografting procedures. For instance, Peeters’s group proposed to functionalize screen-printed graphite macroelectrodes via three different methods: dip-coating the electrode in a solution of nanoMIPs, drop-casting solutions of nanoMIPs onto the surface, and electrografting nanoMIPs. The first two methods rely on the physisorption process of nanoparticles onto the electrode surface, while for electrografting, the electrodes were initially incubated in a 4-aminobenzoic acid (4-ABA) solution. Following CV, they were functionalized with benzoic acid. The carboxyl group was then activated through incubation with a solution of EDC and NHS, and nanoMIPs were anchored by drop-casting. The authors noted that when nanoMIPs are only physisorbed onto the surface, they are prone to desorption, limiting the reproducibility of the sensor [107]. This approach has been utilized by the same group for the development of sensors for the detection of SARS-CoV-2 [108] and lysozyme [109].

### 3.2. NanoMIPs within the Electrode Material

Another approach involves incorporating nanoMIPs directly into the electrode material. For instance, this strategy is possible when graphite-based electrodes or carbon paste electrodes are used, as nanoMIPs are mixed with the components assembled to prepare the working electrode, as described in relevant reviews [110,111]. In several examples, multiwalled carbon nanotubes (MWCNTs) [112] or graphite powder [113,114] are mixed with nanoMIP and used as modifiers of a carbon paste electrode. In a typical procedure, graphite powder and/or MWCNTs are mixed with nanoMIPs, homogenized in mortar, and then combined with paraffin oil, serving as a binder. The resulting material is packed into a mold (e.g., a Teflon tube or a capillary) to create a nanoMIP-functionalized carbon paste electrode [35,112,113,114].

Motaharian et al. suggested the use of both imprinted nano- and microparticles to prepare modified carbon paste (CP) electrodes for detecting diazinon pesticide in water and fruit juices [35].

An interesting example is provided by Piletsky’s group [115], which developed an electrochemical sensor for cocaine detection based on nanoMIPs integrated into electrode material. Cocaine-imprinted nanoMIPs obtained by solid-phase synthesis were added to a solution prepared by dissolving PVC and potassium tetrakis(4-chlorophenyl)borate (kTpBCl). The mixture was sonicated and left to dry to obtain a conductive membrane due to the presence of the electroactive kTpBCl. A 7 mm diameter disk was obtained from this membrane and placed inside an ion-selective electrode (ISE) to obtain a potentiometric sensor for cocaine. The sensor response was based on the specific recognition and binding of charged cocaine molecules with the membrane receptors, which subsequently generated a potential difference across the membrane. Similar approaches have been used by other authors [116].

The integration of nanoMIPs within the electrode material is a simple approach that ensures robust nanoparticle immobilization. However, a low amount of nanoMIPs available for target interaction can result due to their possible sinking within the electrode material, thus affecting binding efficiency and kinetics. In addition, sensor reproducibility could be compromised by the scarce control of the procedure used for assembling the electrode, which basically consists of mixing different components without specifically interacting among them [86].

## 4. Generation of Electrochemical Signals in NanoMIP-Based Electrochemical Sensors

When nanoMIPs are used in electrochemical sensing, the electroanalytical signal can be generated directly from the target in the case it is electroactive or indirectly, monitoring the redox processes of an external probe influenced by nanoMIPs binding with the target [117]. Alternatively, the use of electroactive nanoMIPs has been increasingly proposed during the last few years for the detection of non-electrochemically active molecules by monitoring the electroanalytical signal of the receptor. Table 1 lists some examples of direct and indirect target detection by means of electrochemical sensors based on nanoMIPs.

### 4.1. Direct Detection of Analytes

Direct detection is mostly explored with small molecules, which are not particularly suitable for macromolecules due to their non-electroactive nature or their voluminous structures, reducing the possibility of easy and rapid electron transfer to the electrode surface from their electro-oxidizable/reducible functional moieties [117].

For instance, by exploiting the electrochemical activity of fluoxetine, Alizadeh et al. produced molecularly imprinted nanoparticles combined with graphene and graphite within carbon paste electrodes to develop an electrochemical sensor. Specifically, the authors highlighted the necessity of controlling the amount of nanoMIPs in the carbon paste electrode since an increase in imprinted nanoparticle quantity resulted in higher electrical resistance due to their non-conductive features. The direct detection of fluoxetine was carried out at pH 8 via DPV in the potential range of 0–1 V, exploiting the peak current observed at around 0.5 V, where the secondary amine of the molecule is subjected to an irreversible oxidation process. The sensor showed a linear response in the dynamic range 6 × 10^−9^–1.0 × 10^−7^ mol/L and a detection limit of 2.8 × 10^−9^ mol/L. Moreover, it was able to differentiate the target in spiked plasma samples and capsules with high selectivity and a fast response time [118].

Garcia-Mutio et al. developed a high-affinity nanoMIP-based electrochemical sensor for 4-ethylphenol (4EP) direct detection, exploiting the electrochemical oxidation of the molecule ortho position, leading to the production of quinone [119]. The imprinted nanoparticles were produced by solid-phase synthesis and anchored to the carboxylic groups of a SAM of lipoic acid or 11-mercaptoundecanoic acid on the gold electrode surface via amine coupling chemistry, followed by exposure to ethanolamine for the capping of unreacted groups. The authors observed that the sensor prepared with lipoic acid-based SAM in the absence of the capping agent showed a great peak current response towards 4-ethylphenol oxidation. DPV was then used as a detection technique for the determination of 4EP-increasing concentrations. By applying the potential range of 0–0.5 V, the molecule oxidation occurred at around 0.4 V, and the sensor exhibited a linear response in the concentration range of 0.20–5 mg/L, with a detection limit of 0.068 mg/L, and good selectivity tested against structural analogues of 4EP, which could be oxidized in the same potential range [70].

### 4.2. Indirect Detection of Analytes

NanoMIP-based indirect electrochemical detection of non-electroactive molecules exploiting an external redox probe typically requires a preliminary incubation of the electrode-bearing nanoMIPs with the target solution, after which the redox probe signal is registered. The intensity of such a signal is indeed influenced by target rebinding with the imprinted cavities, as in the case of low or no binding, the redox probe molecules can easily reach the electrode, thus producing characteristic current signals. In contrast, the target binding progressively decreases probe access to the electrode surface, with a subsequent proportional decrease in probe redox signals [117]. Since the electrochemical activity of the redox probe is affected by imprinted cavities filled with analyte molecules, this phenomenon is sometimes referred to as the “gate effect” [120].

The most common redox probes are potassium ferrocyanide (K_4_Fe(CN)_6_) and potassium ferricyanide (K_3_Fe(CN)_6_), often coupled in the anionic redox tool [Fe(CN)_6_]^3−/4−^ [121], and hexaammineruthenium (III) ([Ru(NH_3_)_6_]^3+^) as a cationic redox probe, whose in-situ reduction to hexaammineruthenium (II) is reported as redox couple hexaammineruthenium (II)/(III) [122]. The choice of the specific redox marker basically depends on the nature of the template, considering that the diffusion of the redox probe may be definitely influenced by the accumulation of positive or negative charges in the imprinted cavities upon target rebinding [120]. The use of redox markers is often combined with CV and EIS as widely used transduction techniques for exploring the gate effect, with the former being particularly applicable in the case of appreciable peak currents upon nanoMIP exposure to the probe, which means that easy access of the probe molecules to the electrode through the imprinted cavities is enabled. On the other side, EIS is suitable also when defined peak currents cannot be detected on the nanoMIP-electrode in the probe solution. EIS indeed monitors the variations in resistance at the electrode/electrolyte interface upon nanoMIP-target analyte binding. In this sense, EIS provides an indirect but more general way of generating a suitable electroanalytical signal.

**Table 1:** Examples of direct and indirect target detection by means of electrochemical sensors based on molecularly imprinted nanoparticles.

Analyte	Detection Technique ^1^	NanoMIPs Type ^2^	NanoMIPs Synthesis ^3^	Electrode Material and NanoMIPs Anchoring	LOD ^4^	Ref.
Diazinon	CV SWV	NE	SP	Carbon-paste electrode	7.9 × 10^−10^ M	[35]
4EP	CV EIS	NE	SPS	Gold; SAM of MUDA or lipoic acid, EDC/NHS	0.068 mg/L	[70]
Vancomycin	CV	E	SPS	Glassy-carbon electrode; nafion	83 μM	[90]
Morphine	A	NE	PP	Indium-tin oxide glass; electrosynthesis of PEDOT in the presence of nanoMIPs	0.3 mM	[97]
Diphenylamine	DPV	NE	TPE	Gold; electrosynthesis of PEDOT in the presence of nanoMIPs	5.4 μM	[98]
Cilostazol	DPV EIS	NE	PP	Gold; poly(tyramine)	93.5 nM (DPV) 86.5 nM (EIS)	[99]
Trypsin Glucose Paracetamol C4-HSL THC	DPV	E	SPS	Screen-printed gold electrode; cysteamine, EDC/NHS	0.2 nM 0.4 nM 50 nM 0.1 nM 82 nM	[102]
Trypsin	EIS	NE	LPS; SPS	Screen-printed gold electrode; SAM of lipoic acid	1.06 ng/mL	[103]
Insulin	DPV	E	SPS	Screen-printed platinum electrode; APTES, glutaraldehyde	26 fM	[105]
Trypsin THC	CV C	NE	SPS	Gold; poly(tyramine), glutaraldehyde	10^−14^ M 10^−14^ M	[106]
Lysozyme	EIS	NE	SPS	Screen-printed graphite electrode; electrografting of 4-ABA, EDC/NHS	13 pM	[109]
Cocaine	P	NE	SPS	Ion-selective electrode; membrane of PVC, nanoMIPs, NPOE, kTpBCl	nd	[115]
Pb^2+^	P	NE	PP	(1) IIP-poly(vynil chloride)-coated Pt-wire; (2) IIP-PVC membrane; (3) IIP-PVC-coated graphite electrode; (4) IIP-PVC/polyaniline-coated graphite electrode; (5) IIP-PVC/MWCNTs-coated graphite electrode; (6) IIP-PVC/MWCNTs/PA-coated graphite electrode	3.4 × 10^−10^ M	[116]
Fluoxetine	DPV EIS	NE	PP	Carbon-paste electrode	2.8 × 10^−9^ M	[118]
BSA Trypsin	CV EIS	E	SA	Gold; electropolymerization of PAHN	nd	[123]
Sitagliptin	DPV	E	SPS	Screen-printed platinum electrode; APTES, EDC/NHS	0.06 pM	[124]
Glyphosate	DPV EIS	E	SPS	Screen-printed platinum electrode; APTES or AAPS, EDC/NHS	3.7 pM	[125]
Nonanal	CR	E	PP	Gold; drop-casting of a conductive composite of nanoMIPs and AuNPs	4.5 ppm	[126]
Sarcosine	CV DPV	NE	SG	Carbon-paste electrode	0.38 μM	[127]
Estriol	CV DPV EIS	NE	MP	Glassy-carbon electrode; drop-casting	0.16 μM	[128]
Metformin	DPV	E	SPS	Screen-printed platinum electrode; APTES, EDC/NHS	9 pM	[129]
Paracetamol	DPV EIS	E	SPS	Screen-printed carbon electrode; APTES, EDC/NHS	50 μM	[130]
Glucose	CV	E	MSA	gold; electrodeposition	3 × 10^−2^ M	[131]
Fumonisin B_1_	DPV EIS	NE	SPS	Platinum; electrosynthesis of PPY-(zinc porphyrin), EDC/NHS	0.03 fM (EIS) 0.7 fM (DPV)	[132]
Histamine	P	NE	SPS	Ion-selective electrode; membrane of PVC, nanoMIPs, plasticizer, kTpBCl	1.12 × 10^−6^ M	[133]
Amphetamine	DPV CV	E	SPS	Graphite; chitosan, or chitosan and graphene oxide	0.3 nM	[134]
MDMA	DPV	E	SPS	Screen-printed graphite electrode; nanoMIPs with chitosan and graphene oxide	1.6 nM	[135]
Pb^2+^	DPV	NE	PP	Carbon-paste electrode	30 pM	[136]
Mg^2+^	SWV	NE	TPP	Carbon-paste electrode	0.029 nM	[137]
Cd^2+^	DPV	NE	BCT	Carbon-paste electrode	1.94 nM	[138]
Cu^2+^	DPV	NE	FRP	Screen-printed gold electrode; cysteamine, EDC/NHS	74 pM	[139]
Bisphenol A	P	NE	PP	Paper-based electrode; membrane of nanoMIPs, TDMAC, ETH, PVC, DOP	0.15 μM	[140]
Diazinon	SWV	NE	BP	Carbon-paste electrode	410 pM	[141]

^1^ A = amperometry; CV = cyclic voltammetry; EIS = electrochemical impedance spectroscopy; DPV = differential pulse voltammetry; SWV = square wave voltammetry; CR = chemiresistive; C = capacitance measurement; P = potentiometry. ^2^ E = electroactive; NE = non-electroactive. ^3^ PP = precipitation polymerization; TPP = thermal precipitation polymerization; SA = self-assembly; LPS = liquid-phase synthesis; SPS = solid-phase synthesis; SG = sol–gel; MP = micelle-mediated polymerization; MSA = macromolecular self-assembly; SP = suspension polymerization; BCT = bulk copolymerization technique; FRP = free-radical polymerization; BP = bulk polymerization. ^4^ nd = not declared.

D’Aurelio et al. reported the indirect detection of cocaine by functionalizing screen-printed gold electrodes with molecularly imprinted nanoparticles, which were anchored onto the surface of gold electrodes by means of amine coupling chemistry with an 11-mercaptoundecanoic acid self-assembly monolayer. EIS in [Fe(CN)_6_]^3−/4−^ at 0.12 V was applied to record the sensor response towards increasing concentrations of the target, displaying a linear response in the range of 100 pg/mL–50 ng/mL and a low detection limit of 0.24 ng/mL, making the as-produced sensor applicable for portable and real-time sensing of cocaine [101].

In another example, Zhao et al. fabricated a nanoMIP-based electrochemical sensor for the detection of trypsin and bovine serum albumin (BSA) as dual templates. The electropolymerizable amphilic macromonomer based on acrylamide (AM), 2-hydroxyethyl acrylate (HEA), and N-vinyl carbazole (NVc) (referred to as PAHN, poly(AM-co-HEA-co-NVc) was synthesized and dissolved in DMF with BSA or trypsin. After stirring and dialysis steps, the imprinted nanoparticles with a size of 280 nm were obtained, drop-casted onto the electrode surface, and left to dry to anchor. Finally, electropolymerization was performed to fabricate an electroactive copolymer, where AM acted as a protein affinity moiety, NVc represented the electroactive and electropolymerizable units, and HEA increased the flexibility of the polymer chain. The copolymer was found to be effective in establishing hydrogen bonding and electrostatic interactions with the protein. After polymerization, the authors noted a current increasing for [Fe(CN)_6_]^3−/4−^ due to the formation of an electroactive matrix. The as-produced sensor was used for BSA detection by differential pulse stripping voltammetry (DPSV) in a probe solution, registering a linear response in the concentration range of 10^−14^–10^−5^ mg/mL. Sensor stability, selectivity against competitive proteins, and performance in urine samples were also assessed to prove sensor efficiency for real-time sensing of protein biomarkers. Imprinted nanoparticles were also produced towards another protein target, namely, trypsin, and the resulting electroactive nanoMIP-based sensor showed equally satisfactory analytical performances. The authors noted that the as-produced macromonomer preserved the protein complex structures, while the presence of electroactive units in the resulting electroactive matrix conferred stability to the resulting platform and enhanced electronic transmission [123].

### 4.3. Electroactive Molecularly Imprinted Nanoparticles-Based Electrochemical Sensors for Target Indirect Detection

Electroactive molecularly imprinted nanoparticles represent a valid strategy for the indirect detection of non-electroactive molecules.

**Figure 5:**
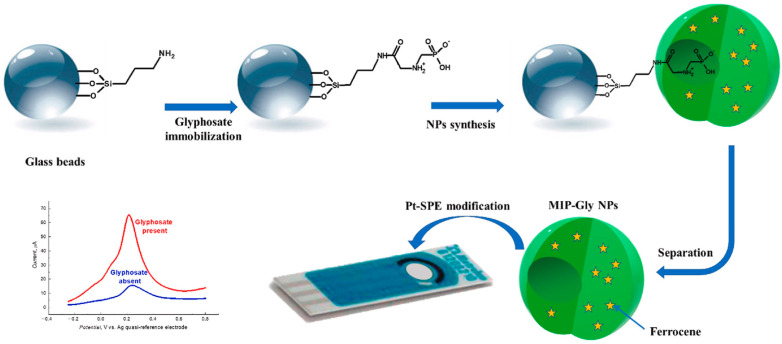
Development of an electrochemical sensor for glyphosate indirect detection based on redox-active nanoMIPs produced in the presence of ferrocene. Adapted from [125].

Mazzotta et al. reported for the first time the pioneering production of imprinted nanoparticles by solid-phase synthesis, where two electroactive ferrocene derivatives, namely, vinylferrocene (VF) and ferrocenylmethyl methacrylate (FMMA), were added in the polymerization mixture. The presence of the electroactive ferrocene-based monomers eminently conferred electroactive properties to the as-produced nanoMIPs having a size of about 160–360 nm, which were able to detect a non-electroactive target, vancomycin, without the need of a redox probe. The nanoparticles prepared with FMMA showed incredible electroactivity, suggesting the total incorporation of the ferrocene moieties in the polymeric structures, further corroborated by XPS analysis. In contrast, VF displayed negligible electrochemical signals due to its poor incorporation in the polymeric structure. The imprinted nanoparticles were then anchored on a Nafion-modified glassy carbon electrode through a self-assembly process, showing the well-defined peaks on CV curves and a decreased formal potential as a result of the improved electron transfer process. The vancomycin detection was indirectly performed by incubating the as-produced sensor with increasing concentrations of the target. As a result, anodic and cathodic peak currents decreased proportionally to vancomycin concentration due to the fact that the target rebinding progressively impeded counterions from accessing the redox molecule; therefore, the electron transfer was definitely hindered. The resulting platform exhibited a linear response in the concentration range of 83–410 µM with a detection limit of 83 µM, which is lower than the recommended plasma concentration range [90]. This study paved the way for an elegant and innovative detection of non-electroactive target analytes, which was replicated by other groups, as reported for the electrochemical indirect detection of sitagliptin, a hypoglycemic agent that is used for the reduction of blood glucose levels in diabatic patients. Sitagliptin-imprinted nanoparticles were produced by solid-phase synthesis in the presence of ferrocenylmethyl methacrylate and then attached to the surface of a platinum electrode by amine coupling chemistry. By applying DPV as an electrochemical technique, when the analyte interacted with the imprinted cavities, a current increase was observed at −0.1 V, which was totally attributed to the ferrocene moieties since sitagliptin oxidation was not observed in the working potential range. Therefore, the interaction of sitagliptin with the imprinted cavities caused a polymer swelling that allowed ferrocene molecules to be exposed at the particle interface. This polymer conformational change was found to be directly proportional to the sitagliptin concentration. Indeed, the sensor was able to detect the target in the concentration range of 100–2000 pM with high sensitivity and a low detection limit, proving the potential of the as-produced sensor for point-of-care diagnosis [124]. Another example was reported by Lach et al. for the detection of the herbicide glyphosate, which emerged as a pollutant due to its widespread use and high water solubility. The authors produced redox-active glyphosate-imprinted nanoparticles (MIP-Gly-NPs) that were integrated on a screen-printed platinum electrode for the fabrication of an electrochemical sensor aiming at selective glyphosate detection in water (Figure 5). During the solid-phase synthesis of nanoMIPs, ferrocene was added into the polymerization mixture, and, after the anchoring of MIP-Gly NPs through saline-glutaraldehyde covalent linkage onto the electrode, the voltammogram clearly displayed the ferrocene oxidation peak at about 0.20 V, further confirmed by EIS measurements. When the as-functionalized electrode was put in contact with glyphosate, a CV current peak increase was observed, along with an impedance decrease, as evidenced by EIS. According to the authors, the target rebinding induced a conformational change of the polymer, which determined the presence of a higher number of ferrocene moieties in the electric double layer at the interface, with the possibility of exploiting such phenomena for generating a proportional and reproducible electroanalytical signal. Indeed, the sensor was able to detect glyphosate in the concentration range of 25–500 pM with a detection limit of 3.7 pM in spiked river water samples with higher selectivity [125].

These examples remarkably show how the simple introduction of ferrocene-bearing molecules in the monomer composition for nanoMIP synthesis can be highly beneficial as it avoids the tedious incubation step of nanoMIPs with target solutions, therefore definitely changing the game. More importantly, target detection is carried out without the influence of a redox probe and can be performed in any medium, significantly expanding the application field of nanoMIP-based electrochemical sensors, with possible use for in situ and in vivo investigations.

## 5. Applications of NanoMIP-Based Electrochemical Sensors

### 5.1. Non-Invasive Diagnostics

Advancements in medical sciences urged the demand for the development of miniaturized sensing tools with optimum selectivity and sensitivity for non-invasive diagnosis of perilous diseases through the detection of appropriate biomarkers in biological fluids. The combination of nanoMIPs with electrochemical transducing systems has enabled the development of pioneering sensing methodologies for point-of-care diagnostics [142].

Interesting studies regarding the use of nanoMIP-based electrochemical sensors for the detection of cancer biomarkers have been reported. Sheydaei et al. fabricated an electrochemical sensor for prostate cancer biomarker sarcosine detection in urine samples using carbon paste electrodes modified with sarcosine-imprinted nanoparticles. Differential pulse voltammetry was performed in sarcosine solutions at different concentrations, and the analyte oxidation signal was recorded in the potential range of −0.4–0.7 V with an anodic peak potential at around 0.5 V. Moreover, it was possible to selectively detect the target in buffered urine samples in the concentration range of 5 µM–1.1 mM, with a detection limit of 0.38 μM [127].

NanoMIPs against trypsin as a pancreatic cancer biomarker and tetrahydrocannabinol were produced by Canfarotta et al. and then anchored onto the surface of gold electrodes, exploiting an electrosynthesized polytyramine film whose amine groups were functionalized with glutaraldehyde to develop a capacitive sensor that was able to selectively detect both targets within the nanomolar range. Additionally, the sensor showed excellent reproducibility, as the prepared electrode was used more than 70 times without any considerable change in sensing performance [106].

An uncommon approach was proposed by Korol et al. for the synthesis of a conductive nanoMIP-based electrochemical sensor for the detection of breast carcinoma biomarker estriol using pristine and derivatized pyrrole monomers. Micelles were synthesized using poly(maleic anhydride-*alt*-1-octadecene) and bis(hexamethylene)triamine; afterwards, pyrrole monomer and template (estriol) were added to the prepared micelles, and polymerization was initiated by adding an aqueous solution of iron(III) p-toluene-sulfonate that resulted in the formation of PPy nanoparticles around the template due to weak interactions between estriol and pyrrole. Derivatized pyrrole nanoparticles were also prepared with the same methodology using 3-(1*H*-pyrrol-1-yl)− 1-propanamine (Py-NH_2_) as a monomer. The solution of nanoparticles was drop-casted onto the electrode surface, and electrochemical characterization was performed by CV. Binding experiments of both MIP and non-imprinted nanoparticles (synthesized without template) prepared with pristine pyrrole and derivatized pyrrole were assessed, showing that pristine pyrrole-based nanoparticles had a higher binding capacity with an imprinting factor of 4.2, while nanoparticles prepared with derivatized pyrrole had no imprinting. The sensor was able to detect estriol within two concentration ranges, namely, 0.5–5 μM and 10–100 μM, with a detection limit of 0.15 μM. The sensor also proved selective against structural analogues of estriol, estrone, and β-estradiol [128].

Manesh et al. fabricated a chemiresistive sensor for the detection of nonanal in human plasma, which is a saturated fatty acid whose concentration in the breath is higher for lung cancer patients compared to healthy individuals [143]. In the study, nanoMIPs were combined with gold nanoparticles to form a conductive composite that was drop-casted onto interdigitated electrodes (IEs). As a result, the target rebinding within the MIP cavities resulted in a conductance change. The sensor was able to selectively detect nonanal in the headspace of human plasma samples in the linear range of 2.5–100 ppm, with a detection limit of 4.5 ppm at room temperature [126].

Drugs represent another class of targets for which non-invasive detection and screening are highly required with the use of rapid, robust, and selective sensing devices. In this context, the integration of nanoMIPs with electrochemical transduction has been successfully proposed. Alanazi et al. fabricated a disposable electrochemical sensor based on nanoMIPs produced in the presence of ferrocene as a redox label for paracetamol detection and screening in human plasma. The attachment of nanoMIPs onto the electrode was performed by covalent bonding through carbodiimide cross-linker chemistry, which resulted in the formation of a uniform layer on the electrode surface. The sensor performance was assessed by differential pulse voltammetry (DPV), resulting in the target detection in spiked plasma samples in the concentration range of 0.1–1 mM, with a limit of detection and quantification of 50 and 167 µM, respectively. The sensor also exhibited higher selectivity, reproducibility, 90 days of shelf life, and a rapid response time of 8 s, which evidenced its capability for point-of-care screening [130].

A smart nanoactuator based on nanoMIPs was developed to detect the anti-diabetic drug metformin in human plasma. Computational studies were performed for the selection of the most suitable monomer and the composition of the polymerization solution. Ferrocene as a redox marker was used for conferring electroactive properties to nanoMIPs, which were then anchored onto the electrode surface by amine coupling chemistry. The metformin detection was performed by DPV, enabling a sensitive response to the drug in plasma solutions within a concentration range of 100 to 2000 pM. A low detection limit of 9 pM was obtained, while no cross-reactivity towards paracetamol and sitagliptin as competitive molecules was observed. The sensor also showed good stability for up to 120 days and reusability [129].

Jyoti et al. developed a nanoMIP-based chemosensor for the detection and quantification of cilostazol (CIL) and 3,4-dehydrocilostazol in human plasma. CIL is a cyclic nucleotide phosphodiesterase inhibitor that has vasodilatory, antimitogenic, and antiplatelet effects and is used to treat intermittent claudication. The imprinted nanoparticles were immobilized on gold disk electrodes by sedimentation, and electropolymerization of tyramine was performed for further stability while immobilizing nanoMIPs on the electrode. Experimental results revealed that acrylic-based nanoMIPs exhibited higher CIL binding capacity than itaconic acid (IA) and 4-VP (4-vinylpyridine) and its derivative, leading to higher selectivity for the target molecules against competitive molecules such as glucose and cholesterol. The sensor was able to detect CIL with high sensitivity in the dynamic concentration range of 134 nM–2.58 μM, with a detection limit of 93.5 and 86.5 nM for DPV and EIS, respectively. Different isotherm models were compared, namely, Langmuir, Freundlich, and Langmuir-Freundlich, revealing the relative homogeneity of imprinted cavities [99].

**Figure 6:**
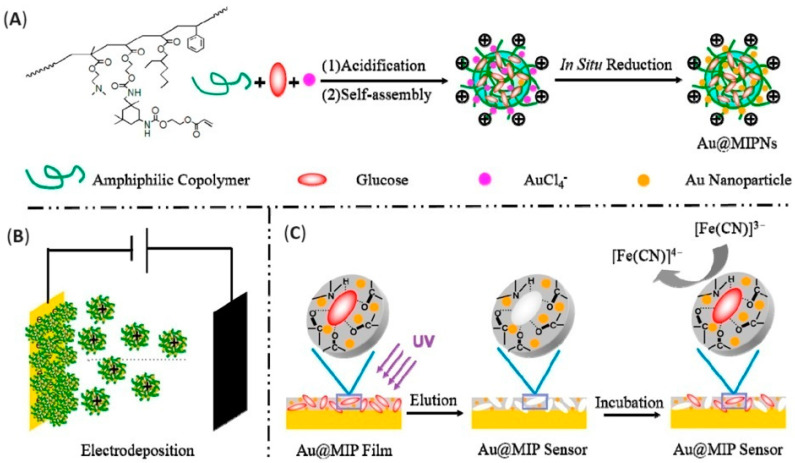
Development of an electrochemical sensor for glucose detection. (**A**) Sensor fabrication. (**B**) Electrodeposition. (**C**) Au@MIP sensor fabrication. Adapted from Ref. [131].

Zhao et al. fabricated an electrochemical sensor based on water-compatible imprinted nanoparticles for glucose monitoring in urine samples (Figure 6). In this study, a copolymer constituted by (dimethylamino)ethylmethacrylate (DMA), 2-hydroxy ethylacrylate (HEA), 2-ethylhexyl acrylate (EHA), and styrene (St), referred to as poly(DMA-*co*-EHA-*co*-HEA-*co*-St), was synthesized and self-assembled around glucose, resulting in imprinted nanoparticles. Gold nanoparticles were used to decorate the nanoMIPs, and the labeled, imprinted nanoparticles were electrodeposited onto gold electrodes. The obtained nanomaterial was subjected to a cross-linking step by UV irradiation, resulting in a stable three-dimensional matrix around the template, which was then removed to create the imprinted cavities. CV was used for monitoring steps during sensor fabrication and the detection of analytes. Two detection ranges were obtained, namely, 10^−10^–10^−8^ mol/L and 10^−8^–10^0^ mol/L, with a detection limit of 3 × 10^−12^ mol/L [131].

The protein hormone insulin is directly involved in the metabolism of glucose. Cruz et al. proposed an electrochemical sensor labeled with the redox probe ferrocene for the detection and quantification of insulin in plasma. DPV was used for monitoring sensor response towards the analyte, showing good detection ability in the concentration range of 50–2000 pM and an extremely low detection limit of 26 and 81 fM in neutral buffer and human plasma, respectively. Moreover, the sensor showed adequate reproducibility for 20 measurements and storage ability up to 168 days [105].

### 5.2. Food Analysis

Fumonisin B_1_ (FB_1_) is among the most hazardous mycotoxins with strong carcinogenic effects and has been found in a wide range of food products, thus affecting food quality and safety. Munawar et al. fabricated an MIP-NP-based electrochemical sensor utilizing free radical polymerization for the synthesis of nanoparticles. A platinum electrode of 1.5 mm diameter was used as a working electrode, at which, prior to nanoMIP immobilization, electropolymerization of polypyrrole-(zinc porphyrin) was performed. The conductive film resulted in high conductivity and allowed the stable covalent attachment of nanoMIPs onto the electrode surface through EDC/NHS coupling. The signal was monitored by the EIS and DPV for analyte sensing. The sensor showed a sensitive determination in the concentration range of 1 fM–10 pM, with a remarkably low detection limit of 0.03 fM for EIS and 0.7 fM for DPV, and a high impact factor of 6.28 was obtained by DPV, remarking the suitability of the as-produced sensor for FB_1_ detection in maize [132]. Lysozyme, a low-molecular-weight cationic protein, possessing bactericidal properties due to which it has been extensively used in the pharmaceutical and food industries, is involved in allergic reactions in some humans even in trace quantities, therefore requiring accurate sensing and screening in clinical and food samples. Singla et al. produced high-affinity nanoMIPs for impedimetric and thermal detection of lysozyme in egg white samples. Thermal analysis was performed with the heat transfer method, and resistance in heat transfer between the solid–liquid interface of the electrode surface was monitored. The sensor successfully detected lysozyme in the concentration range of 1 fM–1 μM, resulting in an exceptionally low detection limit of 13 pM for EIS and 1 pM for thermal analysis. However, the EIS technique showed a lower detection time (10 min) as compared to thermal analysis (30 min), with a negligibly low response towards competitive molecules such as troponin and bovine serum albumin. The low cost and simple preparation made the sensor a promising tool for lysozyme detection as a food allergen [109].

Many chemical substances, such as pesticides, insecticides, and herbicides, are commonly used in agriculture and industrial processes, leading to their accumulation in food and eventually in living organisms. Therefore, it is essential to monitor their trace levels in a wide range of food samples [141]. Organophosphate pesticides (OP) are worldwide used for agricultural purposes, but they are highly toxic for bio-organisms, even at trace levels. A nanoMIP-based electrochemical sensor for diazinon detection in fruits and well water was developed by Motaharian et al. The imprinted nanoparticles were grafted on carbon paste electrodes, and square wave voltammetry (SWV) and CV were used for electrochemical studies. The MIP-modified carbon paste electrode exhibited higher target adsorption when compared to the CPE integrated with non-imprinted nanoparticles. The sensor was able to selectively detect diazinon in the concentration range of 2.5 × 10^−9^–1.0 × 10^−7^ mol/L, with a low detection limit of 7.9 × 10^−10^ mol/L. The sensor also successfully detected diazinon in apple fruits, with recovery percentages of 92.53–100.86% [35].

Volatile phenols, especially 4-ethyl phenol (4EP), confer unwanted organoleptic characters to wines or beers known as “Brett character” and can be used as markers for the freshness of beverages. Garcia-Mutio et al. fabricated a high-affinity MIP-NP-based electrochemical sensor for 4EP detection in wines and beverages. A solid-phase synthesis approach was used for the synthesis of MIP nanoparticles of controlled size and diameter. Afterwards, carbodiimide chemistry was utilized for the assembly of MIP-NPs on the gold electrode surface. All the functionalization steps and analyte responses were monitored by CV and EIS techniques. The sensor was able to selectively detect 4EP within a concentration range of 0.24 to 5 mgL^−1^ in the presence of competitive molecules such as 4-vinyl phenol, ethyl ester, and coumaric acid [70].

Histamine is a biogenic amine commonly found in cheese, fish products, and fermented food, although its presence reduces the quality of food. Basozabal et al. fabricated a nanoMIP-based potentiometric sensor for histamine detection in fish and wine samples. An ion-selective electrode (ISE) comprising Ag/AgCl wire was used for the specific detection of histamine cations, assembled by incorporating nanoMIPs in a PVC membrane, whose conductivity was increased upon the addition of the anionic additive potassium tetrakis (4-chlorophenyl)borate (kTpBCl). Different compositions of plasticizer, MIP nanoparticles, and lipophilic salt were used for PVC membrane fabrication, and the most suitable composition was selected for further experiments. The use of high-affinity nanoparticles boosted sensor performance and provided highly sensitive, label-free detection of the analyte in the concentration range of 10^−6^–10^−2^ mol/L with a considerably low detection limit of 1.12 × 10^−6^ mol/L. A rapid response time of just 20 s proved the usefulness of the as-produced sensor in the real-time monitoring of food products [133].

### 5.3. Water Pollutant Detection

NanoMIP-based electrochemical sensors found prominent applications in the detection of trace pollutants in water. Khadem et al. used nanoMIPs as modifiers for the fabrication of carbon paste electrodes to detect diazinon, a widely used organophosphorus pesticide. The modified electrode was prepared by mixing graphite, paraffin oil, nanoMIPs, and MWCNTs. Square wave voltammetry (SWV) was used for electrochemical characterization and monitoring the sensor response by exploiting the target reduction at the electrode surface in an acidic medium. The sensor was able to detect diazinon in the linear concentration range of 5 × 10^−10^–1 × 10^−6^ mol/L with a detection limit of 1.3 × 10^−10^ mol/L. The sensor response towards real samples was tested, and almost similar results were obtained for the detection of diazinon in river water, tap water, and human urine samples without any pre-treatment [141].

Kamel et al. developed a disposable paper-based potentiometric sensor for the detection of bisphenol A (BPA), a frequent environmental pollutant in water. BPA-imprinted nanoparticles were produced by classical precipitation polymerization, while Whatman chromatographic paper was used for the sensor fabrication by painting it with carbon ink for conductive properties. Afterwards, it was covered with an insulating plastic mask to avoid undesirable currents from the surface, and only a small circular part of almost three millimeters was used for sensing. The imprinted nanoparticles were attached to the surface by a simple drop-casting method. A conventional glassy carbon electrode was also modified with the imprinted nanoparticles, and results were compared for optimized sensing performance. The high affinity of the imprinted receptors resulted in a rapid response to analyte concentrations ranging from 0.5 to 13 µM, with a detection limit of 0.15 µM. High selectivity and excellent recovery rates were also obtained. The results were also validated by HPLC, proving the efficiency of the sensor as an efficient tool for rapid and cost-effective detection of BPA [140].

Increasing heavy metal ion concentrations within the environment, particularly in water bodies, is a matter of concern because of their high toxicity, non-biodegradability, and accumulation in the body, leading to serious health disorders. Despite immense progress in the development of MIP-based electrochemical sensors, only a few studies have been reported for nanoMIP-based electrochemical sensors for the detection of heavy metal ions in water [137]. Ardalani et al. fabricated a lead-ion-imprinted nanoparticles (IIP)-based potentiometric sensor for the detection of lead ions in different aqueous media. The nanoparticles were produced in the presence of 2,2′:6′,6″-terpyridine (Terpy) as a metal ligand, resulting in high-affinity nanoparticles possessing a fast rate of sorption and desorption of lead ions, therefore registering a rapid response time. The IIP showed excellent selectivity against competitive ionic molecules or other heavy metals. The sensor was able to detect ultratrace concentrations of lead ions in the concentration range of 5.3 × 10^−10^–1.0 × 10^−1^ M in aqueous samples, with a remarkably low detection limit of 3.4 × 10^−10^ M. The simple fabrication process, high selectivity, and rapid response of the sensor made it applicable for trace ion analysis in water sources [116]. Bojdi et al. produced lead (II) imprinted polymer nanoparticles (IIP-NPs) in the presence of 4-(2-pyridylazo)-resorcinol as a ligand for lead binding. A carbon paste electrode was modified by a composite of IPP-NPs and graphite powder, and differential pulse stripping voltammetry was used as a characterization and detection technique. The detection of ions was performed after open-circuit sorption and reduction of lead ions to their metallic form. The sensor was able to detect lead ions in spiked water samples in the linear concentration ranges of 0.1–10 nM and 10 nM–10 µM, with a detection limit of 30 pM, proving the applicability of the as-produced sensor in lead detection [136]. The same research group also reported a similar strategy for the detection of mercuric ions in water. A carbon paste electrode was modified with a composite of MWCNTs and mercury-ion-imprinted nanoparticles, and anodic stripping square wave voltammetry was used to monitor the analytical response of the sensor. The sensor showed a linear response in the detection range of 0.1–20 nM with a detection limit of 29 pM. The sensor was able to detect Hg(II) ions in real water samples with results comparable to ICP-MS results, demonstrating the satisfactory performance of the proposed device [137]. Samandari et al. produced cadmium-imprinted nanoparticles in the presence of 4′-(4-vinylphenyl)-2,2′:6′,2″-terpyridine as a binding ligand. The IIP-CPE was fabricated by mixing imprinted nanoparticles and graphite powder, using stripping voltammetry for characterization and generation of analytical responses in spiked blood, rice, and water samples. A linear response was obtained for the concentration range of 4–500 nanomoles and a detection limit of 1.94 nM, while an excellent recovery of 95.0% was registered for all real samples [138]. Di Masi et al. produced copper (II)-imprinted nanoparticles in aqueous medium, which were then immobilized on screen-printed gold electrodes previously functionalized with cysteamine. Differential pulse voltammetry (DPV) was used to check the analytical response of the sensor by exploiting the current signal generated from the interaction of Cu^2+^ ions and imprinted film. The sensor exhibited a linear response in the concentration range of 1.9–61 nM with extremely low detection and quantification limits of 74 and 247 pM, respectively. The sensor also proved to be highly selective against competitive metal ions in water, such as CrO_4_^2−^, Zn^2+^, and Ni^2+^ ions, with similar responses in water samples [139].

### 5.4. Drugs for Abuse Testing

Drug abuse poses health and safety hazards, both in the workplace and in the wider community. There is increasing interest in implementing drug-abuse testing programs in several contexts, from athletes to workers in occupations that are considered critical to public safety and health. Also in this thorny field, where highly performing detection systems are required due to the severe consequences of a positive test, a few examples of nanoMIP-based electrochemical sensors have been proposed. Piletsky et al. fabricated a nanoMIP-based sensor for the potentiometric detection of cocaine in blood serum samples. Two different synthesis approaches (chemical polymerization in water and photopolymerization in organic solvent) were utilized for the fabrication of nanoparticles with four different compositions, selected after molecular modeling. Results showed that nanoparticles prepared by organic solvents had a 20 times higher yield than in water and were therefore chosen for sensor fabrication. Ion-selective membranes were used for the attachment of nanoMIPs for assembling a potentiometric sensor measuring changes in potential difference after the binding of charged cocaine molecules. The sensor was able to selectively detect and quantify the analyte in spiked blood serum samples in the concentration range of 1 nM–1 μM [115].

Truta et al. reported the fabrication of a smart portable electrochemical sensor for the on-field detection of 3,4-methyl-enedioxy-methamphetamine, known as MDMA or molly, which is classified as a drug of abuse. Electroactive nanoMIPs, synthesized in the presence of ferrocene to prompt conductive properties, were integrated onto the electrode surface by suspension with chitosan/graphene oxide. CV was used for the electrochemical characterization of the sensor, observing an increase in the electroactivity of the nanoMIPs after analyte rebinding. The sensor also exhibited high selectivity, as no significant change in sensor response was observed after exposure to competitive molecules. The sensor showed an excellent sensitivity of 106.8 nA × μM^−1^, with a detection limit of 1.6 nM and recovery rates of 92–99%, which were confirmed by UPLC-MS/MS spectroscopy. The as-proposed sensor was shown to be useful for real-time detection of MDMA in street samples [135]. The same group developed an electrochemical sensor based on amphetamine-imprinted nanoparticles produced by solid-phase synthesis in the presence of FcMMA. The imprinted nanoparticles showed a diameter of 269 nm and were immobilized onto screen-printed graphite electrodes upon embedding either in chitosan or in chitosan and graphene oxide, which was then electrochemically reduced. The amphetamine detection was performed by exploiting the so-called “induced fit”, consisting of nanoMIPs swelling upon target rebinding, with a subsequent increase in ferrocene redox signals at increasing target concentrations. Both sensor configurations were able to detect amphetamine in the concentration range of 1–250 nM and 1–200 nM for the first and second configurations, respectively, while in both cases, the limit of detection was 0.3 nM. However, the sensor based on nanoMIPs, chitosan, and reduced graphene oxide showed higher sensitivity. The selectivity was tested against MDMA, cocaine, and methamphetamine, resulting in a satisfactory amphetamine determination, while the application of the as-produced sensors on street samples was confirmed by UPLC-MS. Overall, the authors noted a more significant sensor performance when nanoMIPs were combined with chitosan and reduced graphene oxide, since the latter determined an increased electrochemically active surface, thus definitely enhancing the sensor sensitivity [134].

A THC detection system was developed in an interesting study by Garcia-Cruz et al. The authors aimed at proving the versatility of solid-phase synthesis of nanoMIPs and their implementation in electrochemical sensing and fabricated a “generic” sensing platform able to detect different analytes, namely, C4-homoserine lactone (C4-HSL), trypsin, glucose, tetrahydrocannabinol (THC), and paracetamol, by properly modifying nanoMIP composition. Suitable monomers were selected for each analyte by computational studies. Specifically, electroactive nanoMIPs were produced by introducing polymerizable ferrocene derivatives. The immobilization of nanoMIPs was performed through amine coupling chemistry on cysteamine-functionalized screen-printed gold electrodes, and DPV was used for monitoring the sensor response, correlating the signal to the oxidation peak of ferrocene. What is more, high-affinity electroactive MIPs displayed a rapid response time of just 7 min. The as-produced sensors displayed sensitive responses towards each target in analytically relevant concentration ranges, with low limit of detection values. Furthermore, all the sensors showed good selectivity when exposed to competitive molecules of comparable size and properties. Additionally, the low cost, easy fabrication, and high shelf life made the as-produced sensing platforms advantageous for a wide range of applications [102].

## 6. Conclusions

The above overview was intended to show the prominent progress made in the development of electrochemical sensors based on molecularly imprinted nanoparticles as recognition elements. The most common synthetic strategies for MIPNPs were elucidated, paying particular attention to the innovative solid-phase synthesis that allows for easy and affordable production of MIPNPs. Several methods for the integration of MIPNPs with electrochemical transducers were also taken into consideration, along with the generation of electrochemical signals, depending on the nature of the target. Finally, several applications of MIPNPs-based electrochemical sensors were discussed, highlighting the outstanding performances of such sensing devices.

A high degree of maturity has been reached by the imprinting technology and its scaling down to nanoscale with the synthesis of imprinted nanoparticles, as revealed by the high control of the achievable size and by the different synthetic approaches that can be tailored to the specific requirements related, for instance, to monomers and template solubility and to the nature of the template (biomacromolecules or small/synthetic molecules). Also, nanoMIPs application in electrochemical sensors has experienced a tremendous increase during the last few years, promoted by the development of protocols for easily and reliably anchoring nanoMIPs to the electrode surface and by the demonstrated possibility of using such sensing devices for the detection of both electroactive and non-electroactive compounds by simply modifying the composition of the polymerization mixture.

Despite significant progress documented in the literature, the use of nanoMIP-based electrochemical sensors has not yet met the market, as no commercial applications have appeared so far, with commercially available sensors mostly based on biological counterparts. The reasons for that possibly rely on the costs that are quite high today, at least for large-scale nanoMIP production and, in turn, for the large-scale assembly of nanoMIP-based sensing devices. Another possible limitation in this sense is the lack of general and uniform protocols for integrating nanoMIPs with the electrode and, especially, for hindering a non-specific binding with a suitable blocking agent. This aspect, from one side, increases the versatility of the procedure, but on the other side, it leaves the need for a standardized protocol, which is required for large-scale sensor production. In this sense, preliminary nanoMIP chemical characterization can be highly informative, providing evidence of the availability of specific functional groups that can be used for their coupling with the electrode or can contribute to non-specific binding. Only a few rare examples report information about the characterization of nanoMIPs (e.g., by FT-IR or XPS spectroscopy).

Further enlarging nanoMIP applications to the detection of non-electroactive targets could represent an option for making nanoMIP-based sensing a more widespread technology. As illustrated in the presented overview, this is typically achieved by introducing ferrocene derivatives within nanoMIP structures for monitoring ferrocene redox activity upon target binding as an analytical signal. This approach, which has been revealed to be successful in notable applications, limits the selection of experimental conditions for target detection, especially in terms of applied potentials. As a result, there is low flexibility in selecting the operative detection conditions, which could be required, for instance, to further reduce the possible interference of other co-existing molecules that are electroactive at the explored potential. The introduction of other electroactive probes could further improve nanoMIPs application in sensing devices, allowing for the envisagement of a novel family of highly versatile chemical sensors. Indeed, the possibility of selecting a redox label to embed in the nanoparticle structure further enables the use of nanoMIP-based sensors in any medium, from environmental samples to biofluids, for in situ and in vivo monitoring systems, for which electrochemical transduction is nowadays highly robust and consolidated. This, along with the possibility of reducing costs (e.g., by developing automatic reactors for nanoMIP synthesis) and developing standard protocols for nanoMIP-based electrochemical sensor assembly, can really represent a turning point in the development of this technology, opening wider applications.

## Data Availability

The authors confirm that the data supporting the findings of this study are available within the article.
